# The Role of Plasmid and Resistance Gene Acquisition in the Emergence of ST23 Multi-Drug Resistant, Hypervirulent Klebsiella pneumoniae

**DOI:** 10.1128/spectrum.01929-21

**Published:** 2022-03-17

**Authors:** Pengcheng Du, Chao Liu, Shuaihua Fan, Stephen Baker, Jun Guo

**Affiliations:** a Institute of Infectious Diseases, Beijing Ditan Hospital, Capital Medical University, and Beijing Key Laboratory of Emerging Infectious Diseases, Beijing, China; b Department of Infectious Diseases, Peking University Third Hospital, Beijing, China; c Department of Pulmonary and Critical Care Medicine, Beijing Tsinghua Changgung Hospital, School of Clinical Medicine, Tsinghua University, Beijing, China; d University of Cambridge School of Clinical Medicine, Cambridge Biomedical Campus, Cambridge, United Kingdom; e Department of Medicine, University of Cambridge School of Clinical Medicine, Cambridge Biomedical Campus, Cambridge, United Kingdom; f Department of Geriatric Medicine, Beijing Tsinghua Changgung Hospital, School of Clinical Medicine, Tsinghua University, Beijing, China; National University Hospital

**Keywords:** Hypervirulent *Klebsiella pneumoniae*, IS26, IncFII plasmid, multidrug resistance, sequence type 23

## Abstract

Multidrug-resistant (MDR) hypervirulent Klebsiella pneumoniae (hvKp) sequence type (ST) 23 (MDR-ST23-hvKp) is emerging in China. Despite its increasing importance, this pathogen has not yet been subject to detailed genomic interrogation. We identified 28 ST23 Kp isolated from three hospitals in China. The organisms were subjected to antimicrobial susceptibility testing and whole-genome sequencing (WGS). These novel genomic sequences were analyzed in combination with 218 publicly available genome sequences. We performed molecular serotyping and subtyping, assessed the composition of virulence-associated and antimicrobial resistance (AMR) genes, and determined mobile elements associated with horizontal gene transfer. Two MDR-ST23-hvKp were sequenced by long-read sequencing. The genetic characteristics of MDR and non-MDR isolates were compared. Among the 28 novel ST23 isolates, all were hvKp and 2/28 (7.1%) were MDR-hvKp. From the collection of 246 genomes, KL1 was the predominant serotype (224/246; 91.1%) and the siderophore combination of YbST46-CbST29-AbST1-SmST2 was dominant (101/246; 41.1%); 34/246 (13.8%) organisms belonged to MDR-ST23-hvKp. IncF and IncR plasmid replicons were significantly more prevalent in the MDR group (*P* < 0.05) than in the non-MDR group. IS*26* was commonly involved in AMR acquisition. We observed that the acquisition of AMR genes within the ST23-hvKp was not associated with a loss of virulence genes. A 28-bp fusion site was highly conserved with two copies of the virulence-associated plasmid in ST23-hvKp, and we harbored by some of the IncFII plasmids of MDR-ST23-hvKp. Our data suggest that MDR-ST23-hvKp has undergone multiple independent genetic acquisition and recombination events within different sublineages. Notably, the acquisition of IncFII plasmids and/or IS*26* contributed to the horizontal transfer of AMR genes within ST23-hvKp. Genomic surveillance is essential for further tracking of kMDR-ST23-hvKp.

**IMPORTANCE** Hypervirulent Klebsiella pneumoniae (hvKp) has become the dominant pathotype in hospitals recently. The sequence type (ST) 23 hvKp, which are more commonly associated with the community-acquired infections previously, may have the capacity to acquire multidrug-resistant (MDR) phenotypes creating a new “superbug” (MDR-hvKp) in hospital. In the present study, we studied the associations of MDR and hypervirulence among ST23 K. pneumoniae from our strain collection and publicly accessible genome data. By comparative analysis of the carriage of resistance genes, virulence genes plasmid replicon types, and plasmid sequences, we found that IncFII plasmids were significantly more prevalent in MDR isolates and IS*26* were commonly involved in resistance gene acquisition. We also discovered new MDR plasmids. These results provided an overview landscape of the genetic elements associated with MDR-ST23-hvKp based on currently accessible genome data and calling for further genomic surveillance and well-designed control studies of MDR-ST23-hvKp.

## INTRODUCTION

Klebsiella pneumoniae is a Gram-negative bacterium within the *Enterobacterales*, and a member of the infamous ESKAPE group of pathogens (1, 2). K. pneumoniae is typically a gut commensal, but can also be pathogenic, of which there are two main pathotypes: hypervirulent K. pneumoniae (hvKp) and classical K. pneumoniae (cKp) (3, 4). Typically, cKp are associated with healthcare-associated infections (HAIs) and are commonly multidrug-resistant (MDR). Conversely, hvKp are more commonly associated with community-acquired infections (CAIs) and are generally susceptible to most antimicrobials (5). Previously, hvKp was determined by a positive string test (6). However, the existence of one or more of five virulence genes (*iucA*, *iroB*, *rmpA*, *rmpA2*, and *peg-344*) is now a more accepted definition of hvKp (7). Our previous work suggested that hvKp is replacing the cKp as the dominant pathotype in HAIs in Beijing hospitals (8). Additionally, hvKp organisms may have the capacity to acquire MDR phenotypes creating a new “superbug” (MDR-hvKp) (9, 10). Gu et al. (11) recently reported a fatal outbreak of ST11 carbapenem-resistant hvKp (CR-hvKp) in an intensive care unit in China with a 100% mortality rate. Our previous study indicated that MDR-hvKp is becoming more common in CAI cases in Beijing, proposing that the MDR-hvKp may arise sporadically in hvKp backgrounds (12).

There appear to be two main mechanisms associated with the emergence of MDR-hvKp in China. ST11, an epidemic MDR clone of cKp (13, 14), can acquire virulence genes (15), and alternatively, ST23, a predominant sequence type of hvKp (6), can obtain multiple antimicrobial resistance (AMR) genes (16). A previous study suggested that the former (MDR ST11 acquiring a virulence-like plasmid) was more likely than hvKp transforming into MDR-hvKp (17). However, ST23 hvKp associated with CAIs has had limited attention but is becoming increasingly common (3, 6). Here, aiming to better understand the emergence of MDR-hvKp in China, we studied the genomic characteristics of 28 ST23 hvKp isolated from patients in Beijing hospitals. We combined these contemporaneous sequences with a collection of publicly available ST23 sequences. Our data show that MDR-ST23-hvKp may increase in prevalence in China and multiple independent recombination events contribute to the formation of mosaic virulence and resistance plasmids.

## RESULTS

### The emergence of multiple lineages of MDR-ST23-hvKp.

**FIG 1:**
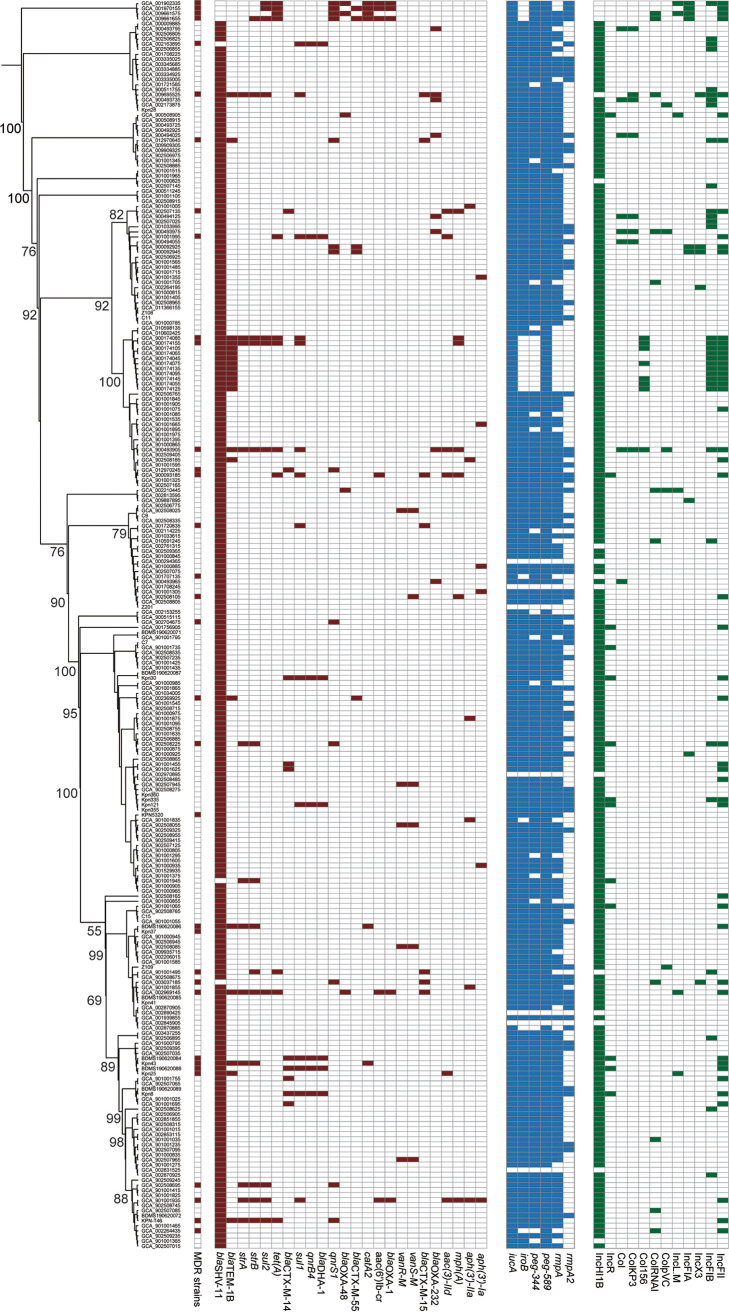
The phylogenetic relationship and carriage of resistance genes, virulence genes, and plasmid replicon types among the 246 genomes enrolled. The phylogenetic tree based on cgSNPs is displayed on the left, and the MDR strains are marked by the bar on the right (red: MDR strains). The carriage of resistance genes, virulence genes, and plasmid replicon types are exhibited by the following heatmaps. The colored block represents the presence of each element.

We contemporaneously studied 28 ST23 hvKp K. pneumoniae, all were isolated in hospitals in Beijing, and two were MDR strains. We accessed and downloaded another 218 public ST23 genome sequences. To investigate the emergence of MDR in ST23 hvKp, we first obtained 31,666 cgSNPs and performed a phylogenetic analysis (Fig. 1). We observed a high degree of genetic diversity among the 246 genomes, which could be distinguished into multiple lineages. Overall, 34/246 (13.8%) were determined to be MDR based on the prediction of resistance genes and the class of antimicrobial agents with which they were associated, and 19/182 (10.4%) were MDR among Chinese strains (Fig. 1, Table S2). A comparable AMR gene profile was observed between the MDR isolates from different lineages. The acquisition of AMR genes in distinct lineages suggests the transfer of these AMR genes on mobilizable genetic elements.

### Molecular characteristics associated with virulence.

**FIG 2:**
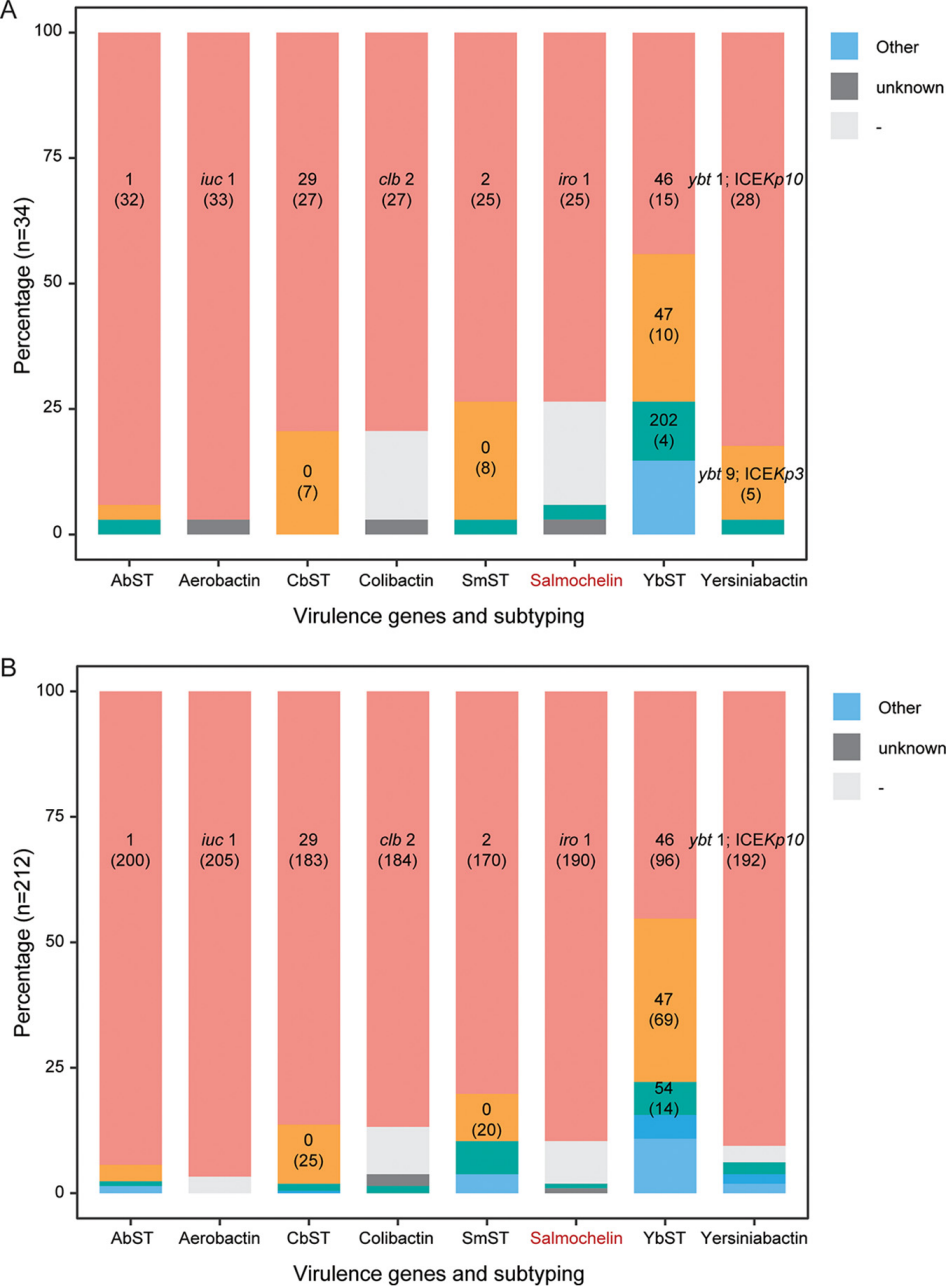
Virulence gene subtypes and plasmid replicons related to resistance among the 246 genomes. The sequence types of gene clusters encoding *aerobactin* (AbST), *colibactin* (CbST), *salmochelin* (SmST), and *yersiniabactin* (YbST), and the corresponding subtypes of the genes, including *iuc*, *clb*, *iro*, and *ybt* locus. (A) MDR isolates. (B) Non-MDR isolates.

From the *in silico* analysis, we found that the KL1 serotype was most common (91.0%; 224/246), the remaining four were KL57. We investigated the presence of the complete coding sequences of *peg-344*, *iroB*, *iucA*, *rmpA*, and *rmpA2* with no frameshift to distinguish hvKp and cKp, 239 were considered hvKp (Fig. 1 and 2). Among other virulence factors, the dominant type of ICEKp (the integrative conjugative element that mobilizes the *ybt* locus encoding siderophore *yersiniabactin* and its receptor) was ICEKp10 with *ybt1*, which was identified in 222/246 (90.2%) of the isolates. The others included six ICEKp3 with *ybt9*, five ICEKp3 with *ybt8*, and a further five ICEKp1 with *ybt2*. The most common subtype of the *clb* gene (encoding *colibactin*) was *clb 2* (85.8%, 211/246). Correspondingly, the most common *iuc* gene (encoding *aerobactin*) was *iuc1* (96.7%, 238/246), and that of *iro* (encoding *salmochelin*) was *iro1* (87.4%, 215/246). Similarly, based on the MLST approach for assigning STs of *yersiniabactin* (YbST), *colibactin* (CbST), *aerobactin* (AbST), and *salmochelin* (SmST), 111 were YbST46, 79 were YbST47, 210 were CbST29, 232 were AbST1, and 195 were SmST2 (all, including the 1 to 3 locus variants of each subtype). In total, 41.1% (101/246) of the isolates belonged to the combination of YbST46-CbST29-AbST1-SmST2, 25.2% (62/246) were YbST47-CbST29-AbST1-SmST2, and 5.7% (14/246) were YbST54-CbST29-AbST1-SmST24 (Fig. 2). In addition, there was almost no significant difference of the dominant types of these genes between MDR and non-MDR organisms, except the *iro1* which was more prevalent among non-MDR isolates (Fig. 2).

### The acquisition of AMR within ST23-hvKp.

Importantly, the acquisition of AMR genes within the ST23 hvKp appeared not to be associated with the loss of virulence-associated genes. We further compared the presence of virulence genes between the MDR and non-MDR isolates (Table S3). Among the five key virulence-associated genes used to distinguish hvKp and cKp, *iucA* and another gene *peg-589*, which is associated with predicting mortality in the sepsis model, were present in all hvKp strains. The carriage of *peg-344* and *rmpA2* in MDR-ST23-hvKp were higher than those in the non-MDR hvKp group, whereas the carriage of *rmpA* in MDR hvKp was lower. The carriage of *iroB* in MDR hvKp was significantly lower than that in non-MDR hvKp (76.5% versus 94.6%, *P* = 0.001). The eight *iroB* negative hvKp isolates belonged to four clusters on the phylogenetic tree, with four, two, one, and one organism in each cluster. Organisms in the first two clusters, including six *iroB* negative hvKp.

### The carriage of plasmid replications, integrative and conjugative elements (ICEs), prophages, and CRISPR/Cas systems in MDR and non-MDR strains.

**FIG 3:**
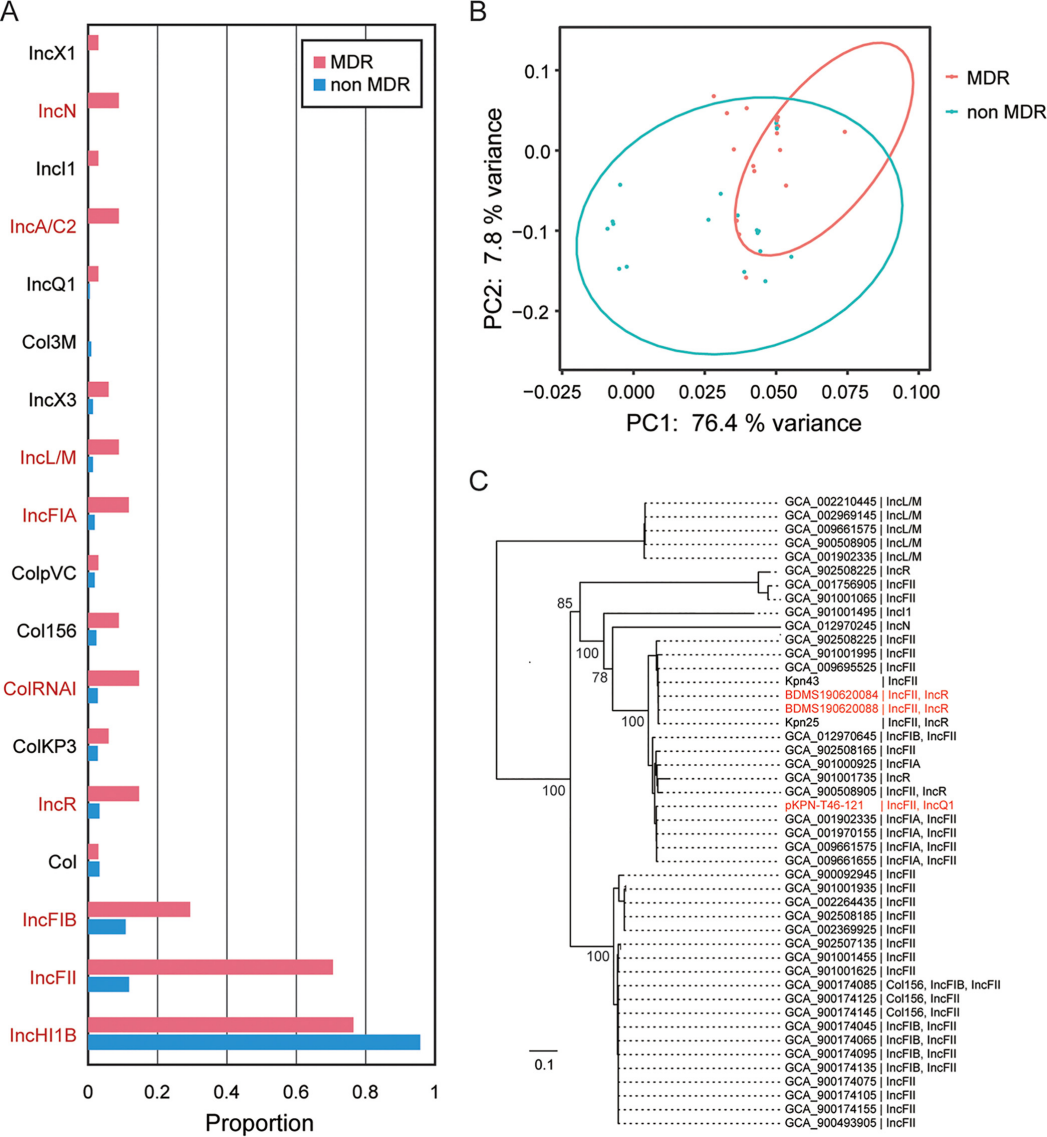
(A) The distribution of plasmid replicons (Inc) among MDR and non-MDR strains. (B) The PCA analysis is based on the carriage of Inc types among the 246 genomes. (C) The phylogenetic analysis of plasmid fragments harbored IncL/M, IncR, Incl1, IncN, and IncF replicons based on the *traI* gene conserved in all enrolled sequences. The three plasmids identified in this study are in red.

To examine the correlation of plasmid-borne elements in the occurrence of MDR ST23 hvKp, we detected the types of plasmids replicons (Inc) and compared the distribution of Inc types between MDR ST23 and non-MDR ST23 organisms. In total, in addition to the IncHI1B type plasmid replicon, which was located in the virulence plasmid of ST23 hvKp, 17 plasmid replicon types were identified, including IncFII, IncFIB, IncR, ColRNAI, IncFIA, IncA/C2, etc (Fig. 3A, Table S4). Among these Inc types, 17 types were found in MDR isolates and 14 were found in non-MDR isolates. IncA/C2, IncN, IncI1, and IncX1 were only found in MDR isolates and Col3M was only found in two non-MDR isolates. Higher diversity of Inc types was found in MDR isolates and the distribution of Inc features was significantly different between MDR and non-MDR organisms (Fig. 3B). The carriage of Inc groups (including IncFII, IncFIB, IncR, and IncFIA) was significantly higher in the MDR isolates than non-MDR isolates (Fig. 3A and B, Table S4).

In addition to plasmids, ICEs and phages are further mobile genetic elements associated with the transfer of AMR genes. We predicted ICEs and prophage sequences and compared the distribution of these elements between sequences from MDR and non-MDR organisms. We identified 2.0 ± 0.6 ICEs (207 ± 105 kb in size) and 1.9 ± 0.6 ICEs (215 ± 94 kb in size) per genome in MDR and non-MDR organisms, respectively. Additionally, the MDR organisms had 7.6 ± 7.4 (195 ± 127 kb in size) prophage per genome compared to 6.1 ± 1.1 (162 ± 31 kb in size) in the non-MDR organisms (Fig. S1A and B). There was no significant difference in the number and total fragment size of predicted ICEs and prophages or the number of AMR genes and ISs located in these elements between MDR and non-MDR organisms. Comparable results were obtained when comparing the composition of CRISPR/Cas systems (Fig. S1C). We observed no obvious between the extent of the core-genome between the MDR and non-MDR isolates. However, with respect to the pan-genome, 1,309 gene groups were significantly more prevalent in MDR organisms compared to non-MDR organisms and 889 gene groups were significantly more prevalent in non-MDR organisms compared to the MDR organisms (Fig. S1D).

### IS*26* closely associated with AMR acquisition in MDR-ST23-hvKp.

AMR genes are often bounded by ISs to form transposons and induce DNA mobility within or between genomes via the conjugation of plasmids or integrative and conjugative elements. Therefore, we predicted ISs in the genomes of MDR-ST23-hvKp and analyzed the relationship of different ISs with resistance genes (Table S5). In total, 20 categories of ISs, including IS*6*, IS*Ecp1*, IS*6100*, IS*903B*, IS*CR1*, and IS*102* were identified. The assembled contigs were further classified as plasmid located ISs or chromosomal ISs, based on their alignment with the Refseq plasmid database. We then aimed to detect any correlation between ISs with AMR genes and plasmid replicon types (Inc). Among the possible plasmid located ISs, we found that IS*26* was the most prevalent IS, associated with several AMR genes and plasmid replicon types (Fig. S2A), and formed groups of common resistance genes, including *bla*_OXA-1_, *bla*_CTX-M-55_, *bla*_TEM-1B_, *aac(3)-IIa*, and *aac(6′)Ib-cr* (Fig. S3). In contrast, fewer ISs were identified in chromosomal, including IS*1400*, IS*1A*, IS*1N*, IS*102*, and IS*Kpn1*, which were associated with a different composition of resistance genes, including *bla*_SHV_, *oqxA*, and *fosA* (Fig. S2B and C).

### The identification of conjugative IncFII plasmids in MDR-ST23-hvKp.

**FIG 4:**
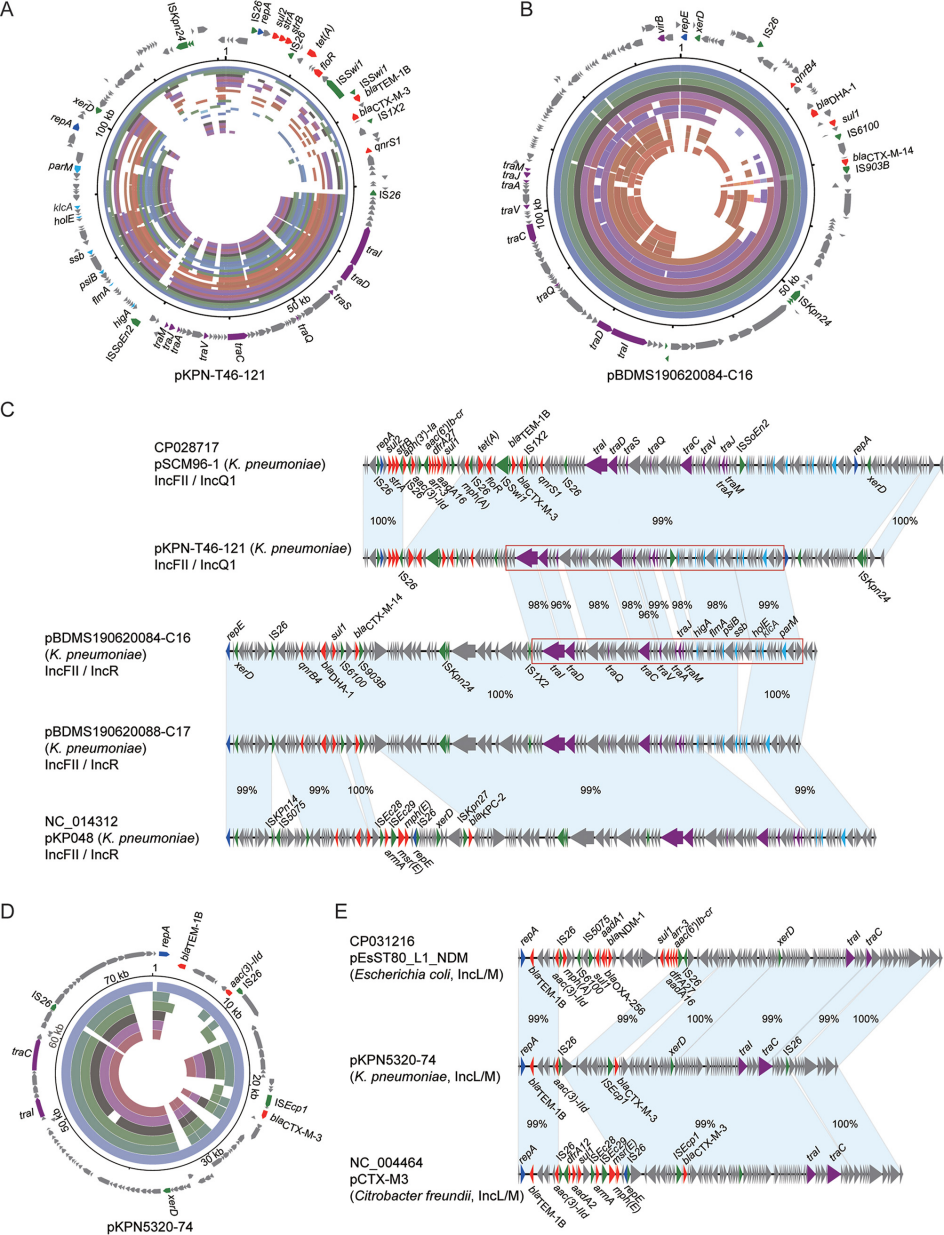
The novel IncFII resistance plasmids identified in this study. (A), (B) and (D) The genetic composition of plasmid pKPN-T46-121, pBDMS190620084-C16, and pKPN5320-74, and the alignments of similar sequences among the 246 genomes. The outer arrows represent the genes related to resistance and transfer (red: antimicrobial resistance; green: integrase recombinase and transposase; purple: transfer associated; dark blue: plasmid replication; gray: other functions). The inner rings distinguished by color represent the alignments of similar sequences among the 246 genomes. (C) The multiple sequence alignments of plasmid pKPN-T46-121 and pBDMS190620084-C16, and the most similar plasmids from the RefSeq plasmid database. The matched regions between two sequences were displayed by light blue blocks and the identities were marked. In addition to the genes related to resistance and transfer, those associated with the plasmid stability are in light blue. (E) The multiple sequence alignments of plasmid pKPN5320-74 and the two similar plasmids from the RefSeq plasmid database.

To further investigate the composition of the AMR determinants and plasmids contributing to the MDR phenotype of ST23 hvKp, we generated the complete genome sequences of the two newly isolated MDR-ST23-hvKp (KpnT46 and Kpn5320, Table S6), identifying two resistance plasmids: pKPN-T46-121 and pKPN5320-74 (Fig. 4A, D, and S). Additionally, we were able to identify two contigs from the draft genomes of organisms BDMS190620084 and pBDMS190620088 that resembled entire resistance plasmids, naming them pBDMS190620084-C16 and pBDMS190620088-C17, respectively. The circularization of the two plasmids was supported by high-quality Illumina reads mapping to both ends of the contigs (Fig. 4B).

**FIG 5:**
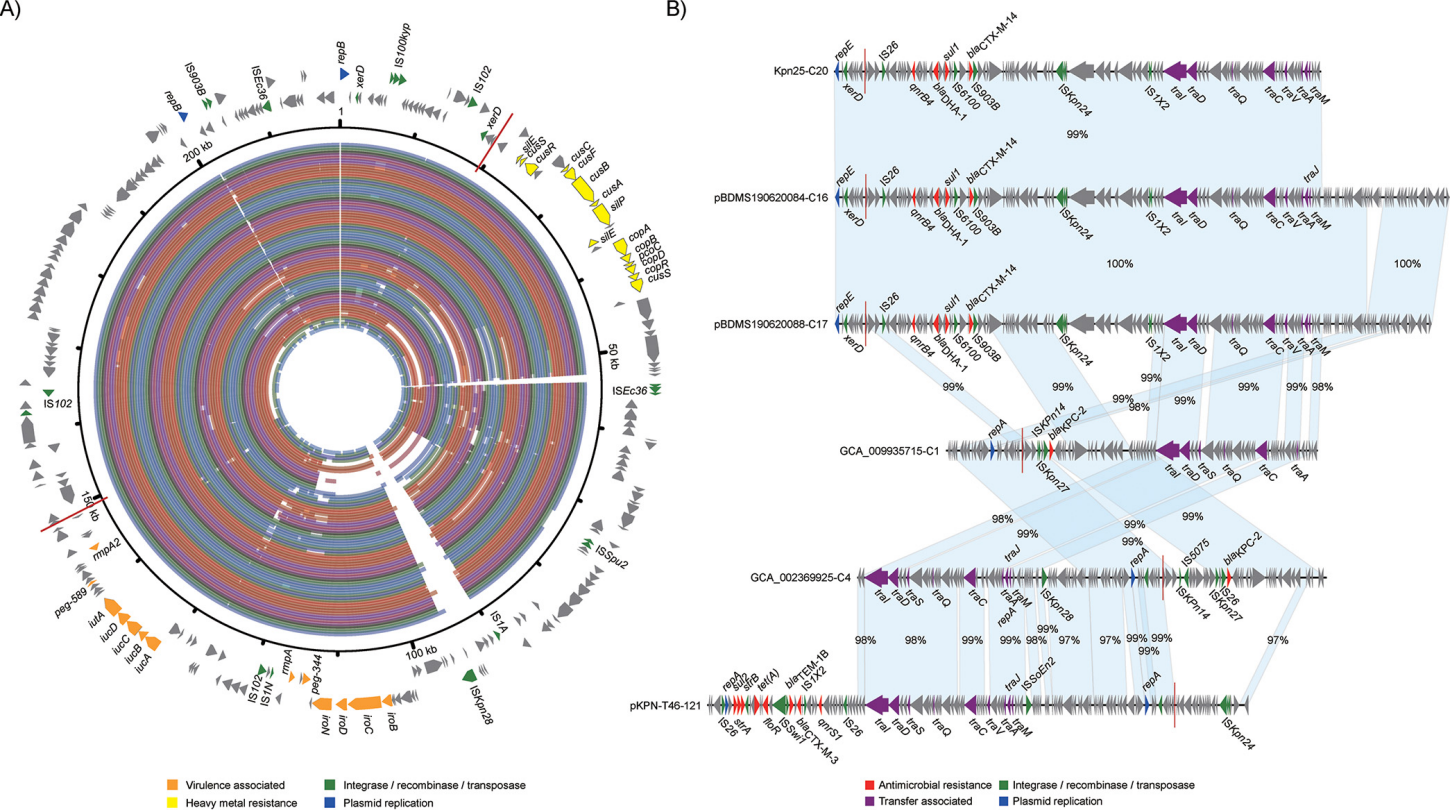
The distribution of 28-bp fusion site in the virulence plasmid and resistance plasmids of ST23 hvKp. (A) The locus of two copies of 28-bp fusion sites in pLVPK, which are marked by red lines. The outer arrows represent the genes related to virulence and heavy metal resistance (orange: virulence; green: integrase recombinase and transposase; dark blue: plasmid replication; gray: other functions). The inner colored circles represent the alignments of virulence plasmids among the 246 ST23 genomes. Among highly similar mapping of different genomes with a total blast score difference <1000, only one result was displayed. (B) The locus of 28-bp fusion sites in resistance plasmids or contigs of resistance plasmids, and the alignments of these plasmid sequences. The locus of 28-bp fusion sites is marked by red lines. The matched regions between two sequences were displayed by light blue blocks and the identities were marked. The arrows represent the genes related to resistance and transfer (red: antimicrobial resistance; green: integrase recombinase and transposase; purple: transfer associated; dark blue: plasmid replication; gray: other functions).

Plasmid pKPN-T46-121 was 121,145 bp with a GC content of 52.3% and comprised both IncFII and IncQ1 replicons, and the *sul2*, *strA*, *strB*, *tet*(A), *floR*, *bla_TEM-1B_*, *bla_CTX-M-3,_* and *qnrS1* AMR genes (Fig. 4C). This plasmid was comparable to the previously described conjugative MDR plasmid (18), pSCM96-1 (accession number: CP028717) from an ST15 K. pneumoniae isolated from sputum in China (Fig. 4C). The key difference between these two plasmids was an ∼15.3 kb insertion in pSCM96-1 encoding eight resistance genes, the remainder had a high synteny and DNA sequence identity (∼99%). Plasmids pBDMS190620084-C16 and pBDMS190620088-C17 comprised the IncFII and IncR replicons, and the *qnrB4*, *bla*_DHA-1_, *sul1*, and *bla*_CTX-M-14_ AMR genes (Fig. 4B). These two plasmids were nearly identical, except for an ∼3.8 kb deletion, and were highly similar (∼99% DNA identity) with the conjugative MDR plasmid pKP048 (accession number: NC_014312) associated with a K. pneumoniae isolated from a patient in China (19) (Fig. 4C). Comparative and phylogenetic analysis showed that plasmid pKPN-T46-121 and pBDMS190620084-C16 represented two clusters of IncFII type resistance plasmids (Fig. 3C and 5C). The conserved sequences of the two assembled plasmids included the *tra* operon encoding the type IV secretion systems (T4SS), and genes involved in plasmid stability and maintenance, including *higA* (antitoxin), *flmA* (stable plasmid inheritance protein), *ssb* (plasmid-derived single-stranded DNA-binding protein), *psiB* (related to plasmid SOS inhibition), *klcA* (anti-restriction protein), and *parM* (plasmid segregation protein) (Fig. 4C).

Plasmid pKPN5320-74 was 74,311 bp with a GC content of 51.9% and comprised the IncL/M replicon, and the *bla_TEM-1B_*, *aac(3)-IId*, and *bla_CTX-M-_*resistance genes (Fig. 4D). This plasmid was comparable to two reported previously MDR plasmids in the RefSeq plasmid collection: pEsST80_L1_NDM (accession number: CP031216) from an Escherichia coli isolated from a patient in the United Kingdom, and pCTX-M3 (accession number: NC_004464) from a Citrobacter freundii isolated from a patient in Poland. The latter was identified as a highly conjugative MDR plasmid responsible for the extensive spread of *bla*_CTX-M-3_ in clinical populations across the *Enterobacterales* (20) (Fig. 4E). The key difference between the three plasmids was two insertions in pEsST80_L1_NDM (∼30 kb) and pCTX-M3 (∼16 kb), respectively, both of which encoded multiple AMR genes. The remainder had a high synteny and identity (∼99%). This plasmid structure was detected in five of the ST23 genomes.

### Multiple conjugative plasmids contribute to AMR acquisition.

To investigate the genetic composition of the plasmids associated with conjugation among this ST23 hvKp, we screened the contigs carrying both plasmid replicons and genes encoding T4SS (Table S7). In addition to the above four resistance plasmids, 47 contigs that could form conjugative plasmids were identified within the 40 genome sequences. Among these contigs and plasmids, 70.6% (36/51) carried the IncFII plasmid replicon. Moreover, these contigs are likely to belong to different types of conjugative plasmids based on the phylogenetic relationships of the *traI* gene conserved in these contigs. Six lineages of *traI* were identified and related to various Inc types, including IncL/M, IncR, IncFII, and IncFIA (Fig. 3C).

Recently, Xu et al. (21) reported that virulence plasmid of ST11 hvKp could transfer via coexisting conjugative IncF plasmids using shared *oriT* regions. Additionally, a specific 28-bp fusion site located in the virulence plasmid of ST11 hvKp could assist with the virulence plasmid forming a hybrid plasmid with conjugative IncF plasmids and then be cotransferred. We screened our ST23 hvKp sequence data for *oriT* sequences and this 28-bp fusion site and found that this site was conserved in virulence plasmids with two copies in each plasmid (Fig. 5A). In addition, among the 51 contigs that may be from conjugative plasmids, six 28-bp fusion sites were found in six contigs of different organisms (Fig. 5B). Moreover, four of the six contigs encoded IncFII plasmid replicons, three encoding an extra IncR replicon, and another encoding an extra IncQ1 plasmid replicon. No shared *oriT* sequence was found in virulence plasmids and resistance plasmids in ST23 hvKp.

## DISCUSSION

MDR-ST23-hvKp is emerging within multiple lineages because of various independent gene acquisition and recombination events comprising various virulence genes and AMR genes within the ST23 hvKp group. We think that MDR-ST23-hvKp is becoming increasingly prevalent and new MDR isolates have emerged because of IS*26* mediated recombination of resistance islands and the horizontal transfer of IncFII plasmids. The genetic context of IS*26* is diverse, which is not just in the AMR or pLVPK plasmid, but also in the chromosome. Notably, ST23 hvKp organisms have acquired various AMR genes but have not lost key virulence-associated genes. Additionally, MDR-ST23-hvKp may have the potential to act as a reservoir for AMR/virulence genes transmission.

A previous study reported that new subtypes of *iuc* and *iro* were widely distributed among *Enterobacterales* (22). Although classical ST23 hvKp mainly carries pLVPK plasmid that commonly presented with the IncHI1B plasmid replications and coharbored *iucA+iroB+peg344+rmpA+rmpA2*, strains that possessed *ybt1-ICEKP-10*, *clb2*, *iuc1*, and *iro1* were the prevalent genotype within the ST23 hvKp group in China, especially in the MDR-ST23-hvKp group (Fig. 1). The previous study showed that the diversity of *ybt* and its associated ICEKp in the chromosome might have resulted from multiple independent varieties (23). Although most of the strains isolated from China carried the *ybt1-ICEKP-10*, the *ybtST* is still diverse, concluding that various microevolution occurred. Among the four virulence genes, *iuc1*, the operon of *aerobactin*, was the relatively conservative gene than others. The subtype of the AbST is sole, which is consistent with a previous study that reported that *aerobactin* is the key virulence gene in the hvKp group (24).

Acquisition of AMR genes was also reported for shaping MDR-ST23-hvKp. IS*26* is highly associated with the transmission of AMR genes among various organisms, as well as hvKp. A previous study reported that *bla*_KPC-2_ (25) and *catA1* (26) have been inserted into the ST23 pLVPK plasmid mediated by IS*26*, which is a relatively unusual way. More importantly, key virulence-associated gene cluster has successfully transmitted into the chromosome by IS*26* within the ST11/KL47 CR-hvKp strains (27). The common way to acquire antimicrobial resistance phenotype was primarily dominated by the acquisition of various antimicrobial-resistant plasmids (28–33). The IS*26* also plays a great role in the formation of resistance plasmids and the insertion of resistance genes into the chromosome (34). It has been reported that ST11 CR-hvKp possessed five tandem repeats of the *bla*_KPC-2_::NTEKPC-Id fragment within the IncR plasmid was also mediated by the IS*26* (35). More importantly, with the help of the IncF plasmids, the nonconjugative virulence plasmid could achieve the mobilization among *Enterobacterales* strains (21). IS*26* was closely related to the IncFII plasmid replication among the ST23 K. pneumoniae, alarming that the transformation and conjugation among these organisms should be emphasized. In this study, we reported two new resistance plasmids from ST23 hvKp isolates by the Nanopore long-read sequencing and found that IS*26* was the most common IS contributed to the acquisition of AMR genes (Fig. S2). Among these MDR-ST23-hvKp strains, we found multiple antimicrobial resistance islands were constructed within the ST23 hvKp group (Fig. S3). Therefore, enhancing the genomic surveillance of the IS*26* and IncF plasmid backbone might be an effective way for monitoring the AMR variation within ST23 hvKp.

For further understanding the reasons for the emergence of MDR-ST23-hvKp, we compared the genomic characteristics between MDR-ST23-hvKp and ST23-hvKp groups. A previous study (17) showed that the plasmid replication load in MDR K. pneumoniae is heavier than that in hvKp and presented with more diversity. In this study, our data also showed that the MDR-ST23-hvKp group possessed more plasmid replication types, concluding that acquiring more antimicrobial resistance associated plasmid and/or fusing with resistance genes within virulence-associated plasmid might be the key. Another concern is the CRISPR/Cas systems. A previous study reported that the absence of type I-E CRISPR/Cas systems in CG258 is strongly associated with the dissemination of IncF epidemic plasmids, triggering the clone to acquire more AMR genes and rapidly spread worldwide (36, 37). However, compared with non-MDR ST23-hvKp, there was no significant difference between the two groups (Fig. S1C), which is similar to Kelly’s study (17). Xu, et al. (21) reported two mechanisms leading to the mobilization of the nonconjugative virulence plasmid of hvKp. The virulence plasmids containing *oriT* sequences identical to those of conjugative plasmids would achieve transfer with the help of the conjugative plasmids. The 28-bp fusion site carried by virulence plasmid and conjugative plasmid could cause fusion of the two plasmids and cotransfer. We have also detected the 28-bp fusion site in virulence plasmid and conjugative plasmid of MDR-ST23-hvKp, indicating that this mechanism might play role in the mobility of the virulence plasmid of ST23-hvKp.

However, due to the incomplete background information of the data from the public database and uncertain representativeness of these strains and genomes, the above differences need further validation by well-designed control studies. In addition, most of the genomes (182/246, 74%) were from China and might introduce selection bias. Therefore, we concluded that the acquisition of AMR-associated genetic elements might be sporadic within the ST23 hvKp. Importantly, it is the MDR-ST23-hvKp that could reserve as a reservoir for AMR or/and virulence genes transmission, and multiple independent recombination events contribute to the formation of mosaic resistance plasmids.

## MATERIALS AND METHODS

### Organisms utilized in this study.

We reviewed a collection of 368 K. pneumoniae isolated from patients in three Beijing hospitals between 2008 to 2020. All organisms were stored at −80°C and identified using MALDI-TOF bacterial identification systems. ST23 K. pneumoniae were detected by PCR amplification or whole-genome sequencing, as previously described (38). HAIs were defined as infections occurring 48 h or longer after hospitalization. Infections occurring within 48 h of admission were classed as CAIs.

### Antimicrobial susceptibility testing, hypermucoviscousity, and virulence genotyping.

Antimicrobial susceptibility testing was performed using a Vitek 2 system, and the disk diffusion method according to the guidelines provided by the Clinical and Laboratory Standards Institute (CLSI) was used for confirmation when needed (39). MDR organisms were defined as those being resistant to greater than or equal to three classes of antimicrobials (23). HvKp was defined as carriage of at least one of the *peg-344*, *iroB*, *iucA*, *rmpA*, and *rmpA2* genes (7, 8), and the genes encoding *yersiniabactin* (*ybt*), *colibactin* (*clb*), *aerobactin* (*iuc*), and *salmochelin* (*iro*) were also detected for virulence genotyping. The hypermucoviscous phenotype was evaluated by the string test (6, 40).

### Genome sequencing and public data collection.

We subjected all 28 ST23 K. pneumoniae from the three hospitals in Beijing to WGS (Table S1). DNA was extracted by the TIANamp Bacteria DNA kit DP302 (Tiangen Biotech, Beijing, China), libraries were prepared using Nextera technology. Paired-end reads of 150 bp were generated by Illumina HiSeq 2500. Additionally, two MDR-ST23-hvKp were selected for the Nanopore long-read sequencing. Additional K. pneumoniae genomes were accessed from the GenBank database and determined sequence types using Kleborate software (23). All ST23 genomes (218 genomes, accessed on June 30, 2020) were included in downstream analysis, resulting in a total of 246 ST23 genome sequences (Table S1). The majority (94.7%; 233/246) of these genome sequences originated from organisms isolated from humans, 11 were from the environment. Additionally, 74.0% (182/246) of the ST23 sequences were from organisms isolated in China between 2008 and 2020 (Table S1).

### Bioinformatic analysis.

Initially, the raw Illumina sequencing data were filtered to remove low-quality reads by fastQC and then *de novo* assembled using SPAdes v3.13 and annotated by Prokka, as previously described (12, 41). AMR genes, virulence genes, insertion sequences (ISs), and plasmid replicon types were identified by ResFinder (42), Virulence Factor Database (43), IsFinder (44), and plasmidFinder (45) database. The complete genomes of K. pneumoniae ST23 isolate KPN5320 and KPN-T46 were determined by combining high accurate short-read by Illumina sequencing and long-read by Oxford Nanopore Technologies-MinION platforms via *de novo* assembly with a hybrid strategy and assembled using Unicycler (46). AMR and virulence genes were identified using thresholds of 90% DNA sequence identity and minimum length coverage of 80%. Kleborate software was used to determine STs and to detect *Yersiniabactin*, *Colibactin*, *Aerobactin*, *Salmochelin*, of K and O locus serotypes, associated ICEKp, and the number of antimicrobial classes the specific organism was predicted to be resistant to (23). The core- and pan-genomes were determined using Roary (47) via default parameters based on the gff files generated by Prokka.

Sequencing reads were mapped to the K. pneumoniae SGH-10 using bowtie 2 v2.2.8 (48) and single nucleotide polymorphisms (SNPs) were identified by using Samtools v1.9 and combined according to the reference genome (SGH-10) using the iSNV-calling pipeline (https://github.com/generality/iSNV-calling). High-quality SNPs (>5 reads of mapping quality > 20) were retained. Regions of recombination were detected by Gubbins (49), and the polymorphic sites located in recombination regions were removed. The concatenated sequences of filtered polymorphic sites conserved in all genomes (core genome SNPs, cgSNPs) were used to perform phylogenetic analysis using the maximum likelihood method by FastTree (50). By comparing draft genome sequences with the complete genome of SGH10 we first identified chromosomal contigs, then we predicted the integrative and conjugative elements (ICEs) using ICEfinder (51), and predicted prophages using PhiSpy (52). CRISPR/cas systems were detected using CRISPRDetect v2.4 (53) by the default parameters. The *oriT* sequences were predicted by comparison with the oriTDB (54) using BLAST, and the 28-bp fusion site was identified using PatMatch (55).

### Statistical analysis.

To determine if plasmid replicon types, subtypes of virulence genes differed between MDR and non-MDR isolates, we performed Fisher's test in R software (fisher.test function in psych package). *t* tests were performed in R to compare the numbers and total fragment sizes of predicted ICEs and prophages, as well as the numbers of AMR genes and ISs located in these elements between the two groups. All tests were 2-tailed. *P* < 0.05 was considered to be statistically significant.

### Data availability.

The genome sequences sequenced in this study have been submitted to the GenBank database under the accession numbers PRJNA685215 and PRJNA575579.
