# Supplementary material for: The Role of Plasmid and Resistance Gene Acquisition in the Emergence of ST23 Multi-Drug Resistant, Hypervirulent Klebsiella pneumoniae
Source: Microbiol Spectr. 2022 Mar 17;10(2):e01929-21. doi: 10.1128/spectrum.01929-21 (PMC9045268; doi:10.1128/spectrum.01929-21)
Supplement: SUPPLEMENTAL FILE 2 — Supplemental material. Download SPECTRUM01929-21_Supp_2_seq8.pdf, PDF file, 0.9 MB [file spectrum01929-21_supp_2_seq8.pdf]

Figure S1. The distribution of prophages, ICEs, CRISPR system and pan-genome among the genomes. (A) and (B) The numbers and total fragment sizes of predicted ICEs and prophages, as well as the numbers of resistance genes and ISs located in these elements. (C) The type of CRISPR systems predicted among the 246 genomes and the number of related genomes. (D) The carriage rate of gene groups of the pangenome of the 246 strains in MDR (*y* axis) and non MDR (*x* axis) groups. The red circles present the gene groups significantly more prevalent in MDR strains, and the blue circles present significantly more prevalent in non MDR strains.

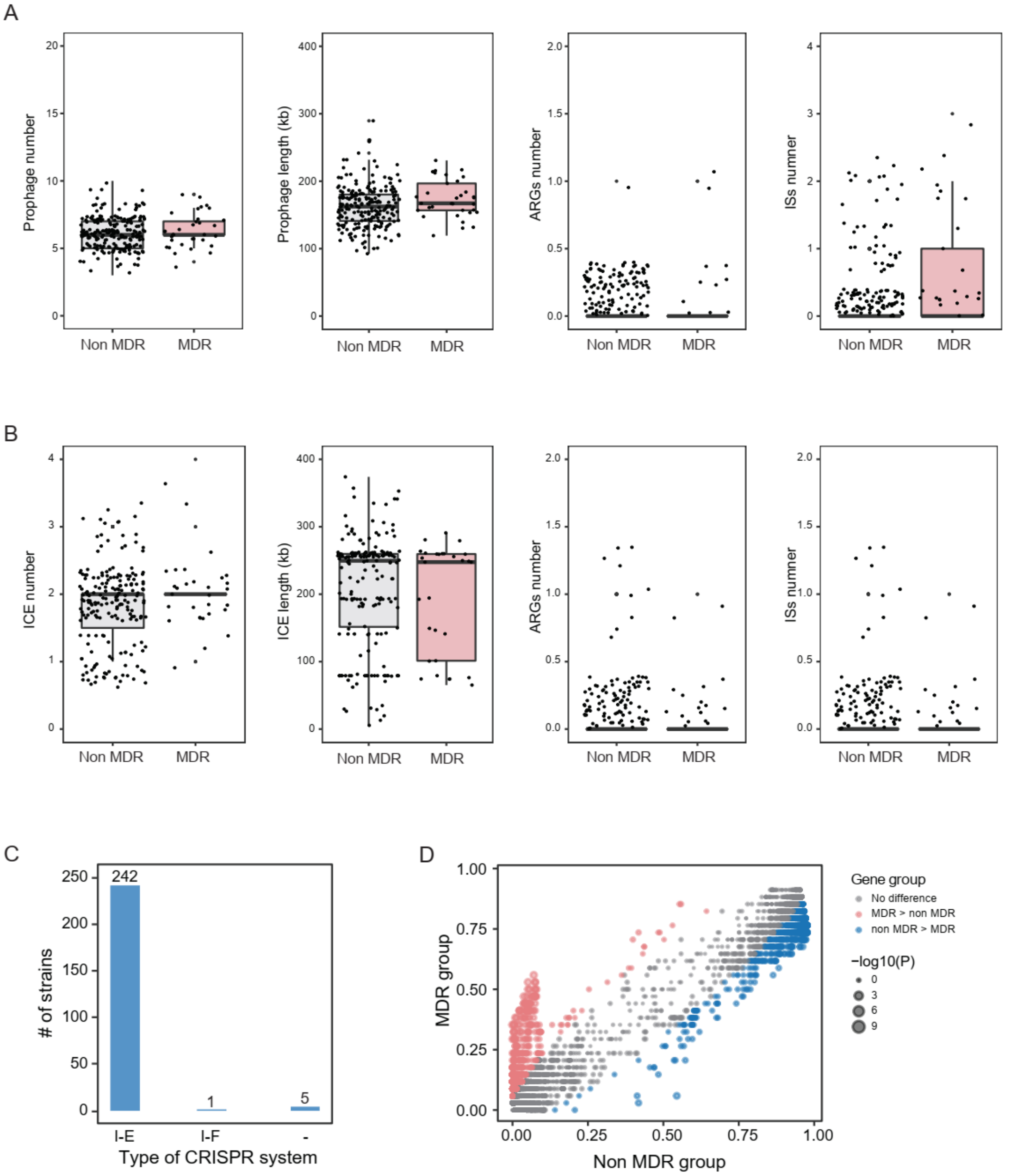

Figure S2. The correlations of plasmid types, ISs and resistance genes in ST23 *K. pneumoniae*. (A) The correlations of plasmid types, ISs and resistance genes in plasmid sequences. (B) The correlations of plasmid ISs and resistance genes in chromosomal sequences.

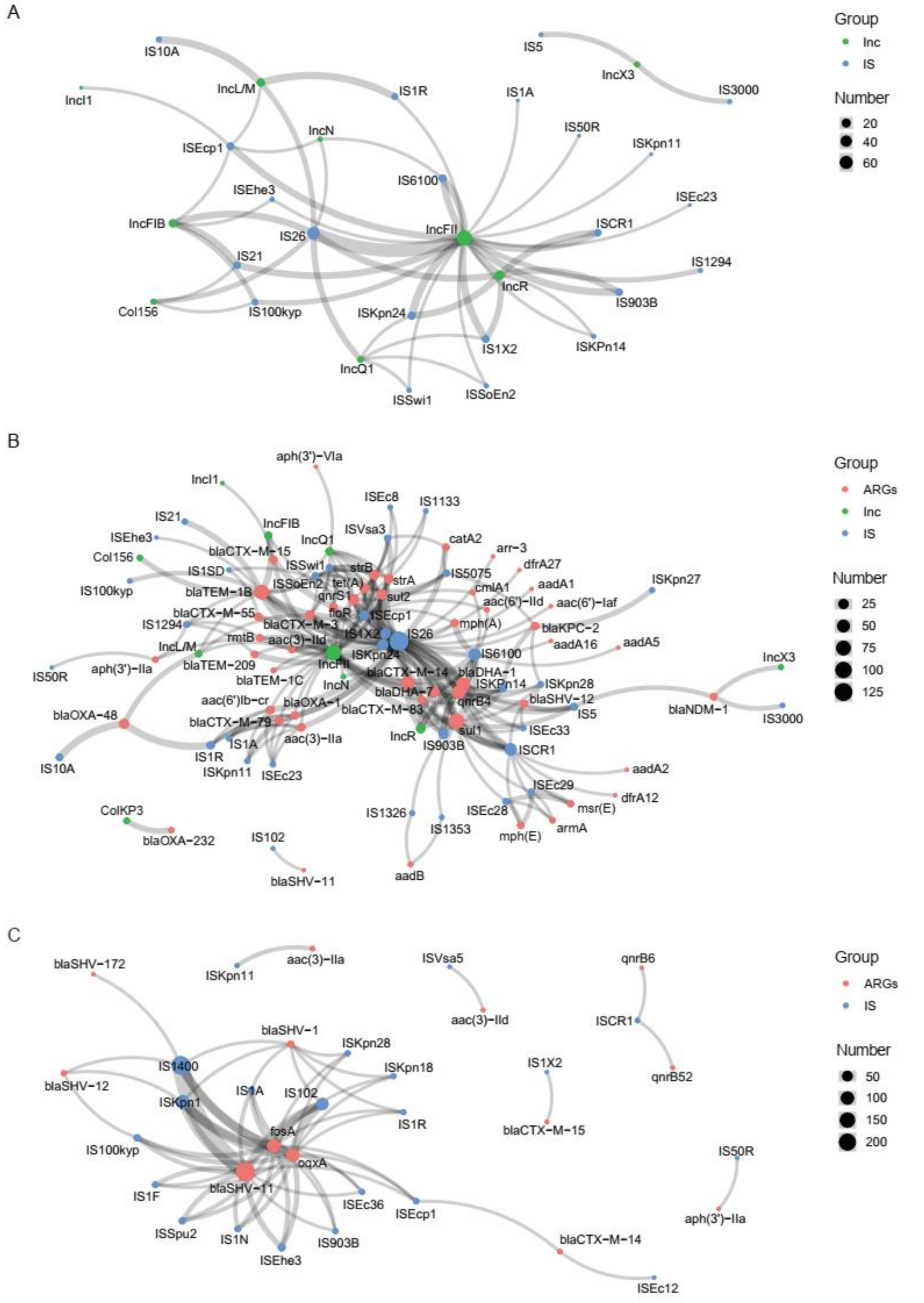

Figure S3. Representative genomic islands encoding resistance genes identified among the 246 genomes. (red: antimicrobial resistance; green: integrase recombinase and transposase; gray: other functions).

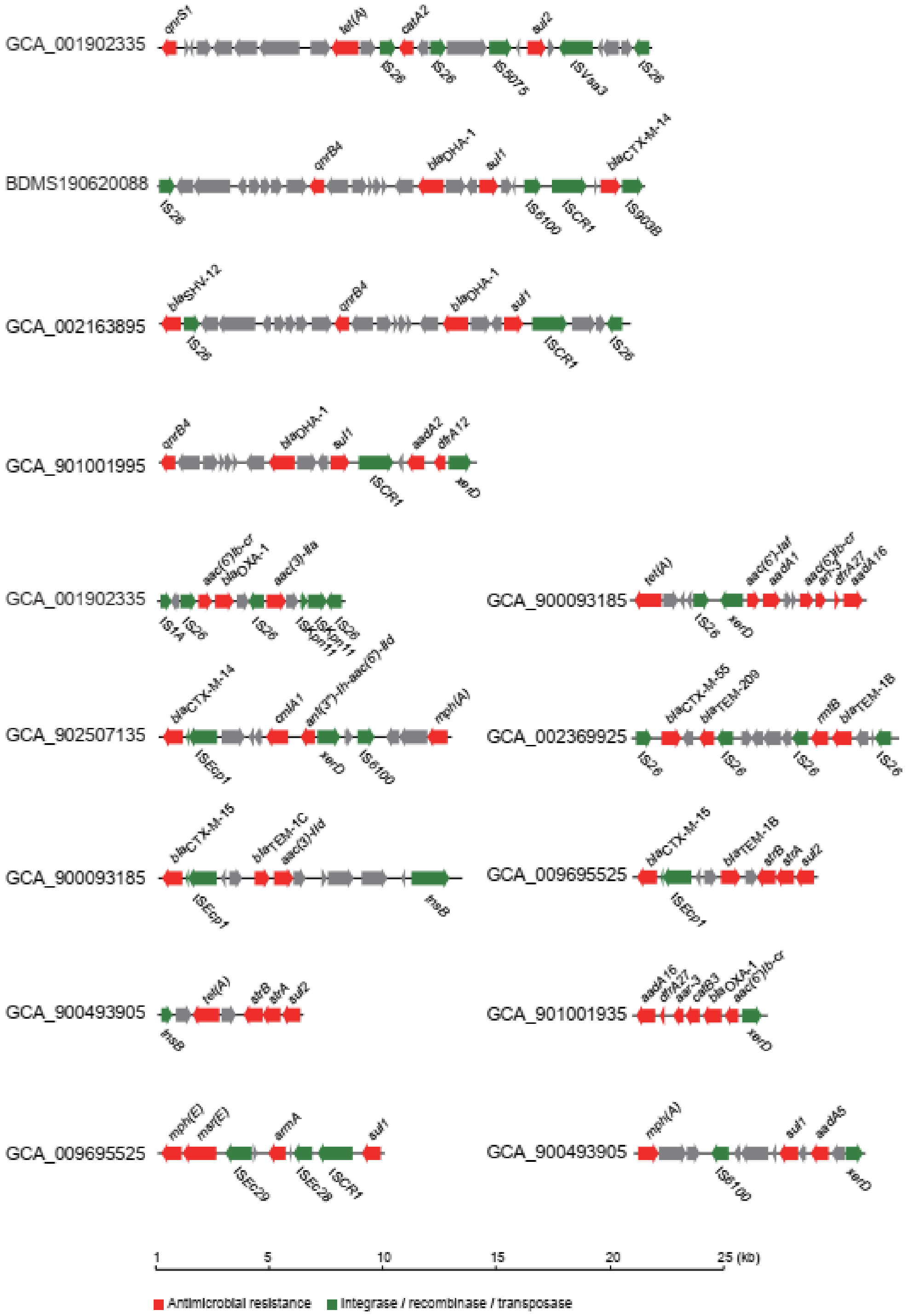

Figure S4. The genetic composition of plasmid pKPN-T46-121 (A), pBDMS190620084-C16 (B), and pKPN5320-74 (C), and the alignments of similar plasmids from RefSeq plasmid database. The outer arrows represent the genes related resistance and transfer (red: antimicrobial resistance; green: integrase recombinase and transposase; purple: transfer associated; dark blue: plasmid replication; gray: other functions). The inner rings distinguished by color represent the alignments of similar plasmids.

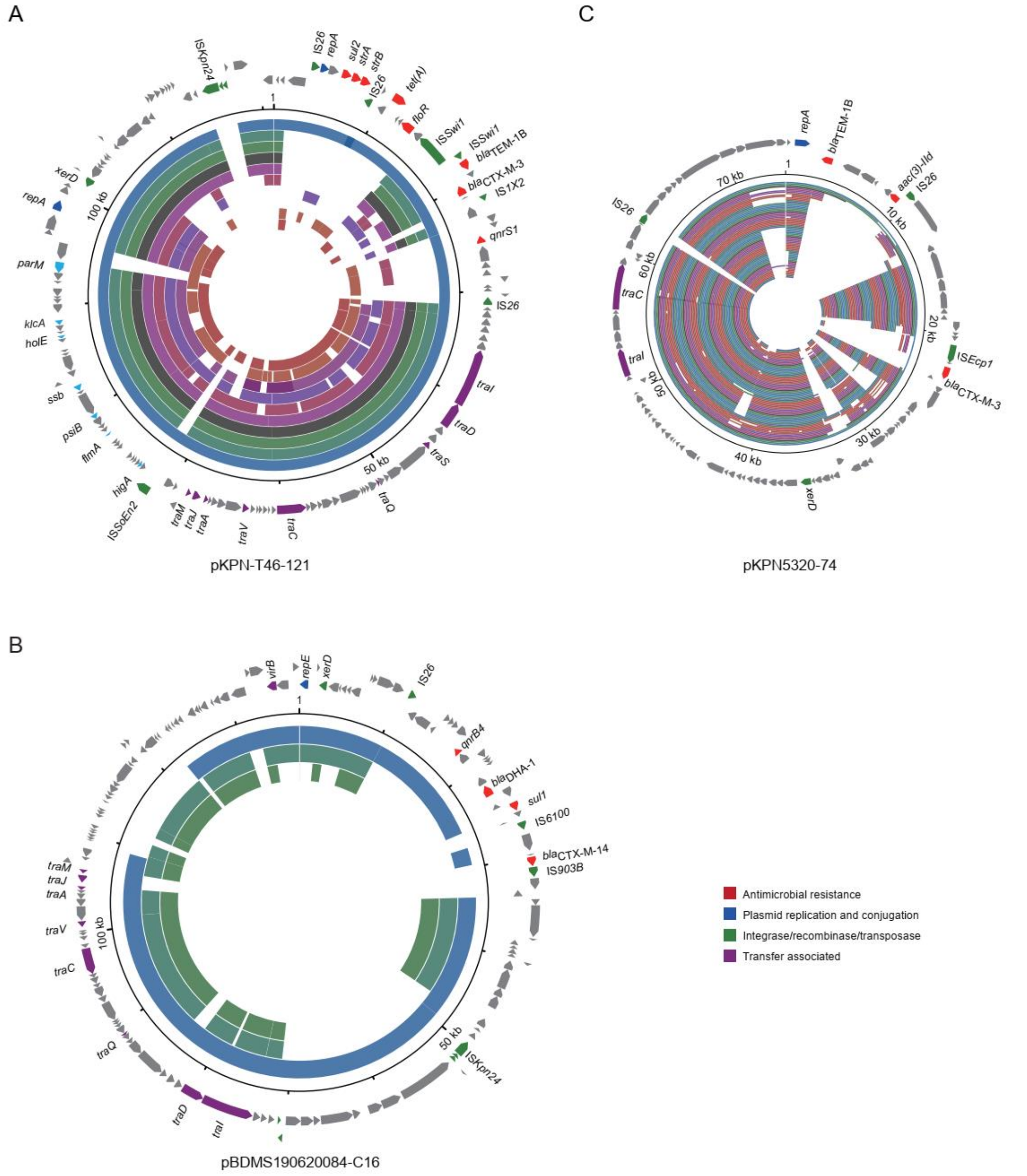

Table S1. ST23 genomes enrolled in this study.

Accession number	Strain name	Host	Source	Geographic location	Collection date
GCA_001902335	Kp_Goe_154414	Homo sapiens	wound swab	Germany: Goettingen	2014/7/21
GCA_001970155	KP254	Homo sapiens	-	Russia: Nizhny Novgorod	Feb-16
GCA_009661575	CriePir140	Homo sapiens	blood	Russia: Moscow	2017
GCA_009661655	CriePir108	Homo sapiens	tracheal aspirate	Russia: Moscow	2017
GCA_000009885	NTUH-K2044	-	blood	-	-
GCA_900493795	4300STDY6470420	Homo sapiens	-	Thailand	2016
GCA_902506805	A5	Homo sapiens	-	China	2008
GCA_902506825	A9	Homo sapiens	-	China	2008
GCA_002163895	RJA166	Homo sapiens	sputum	China: Shanghai	4-May-15
GCA_902506855	B2	Homo sapiens	-	China	2009
GCA_001708225	ED23	Homo sapiens	blood	Taiwan	2006
GCA_003335025	JHTS001	Homo sapiens	hospital, patient	USA: Baltimore	2015/7/27
GCA_003345685	JHTS005	Homo sapiens	hospital, patient	USA: Baltimore	2015/8/28
GCA_003334885	JHTS006	Homo sapiens	hospital, patient	USA: Baltimore	2015/8/28
GCA_003334925	JHTS007	Homo sapiens	hospital, patient	USA: Baltimore	2015/9/4
GCA_003335005	JHTS002	Homo sapiens	hospital, patient	USA: Baltimore	2015/7/29
GCA_001721585	BA253	Homo sapiens	blood	India	2016

GCA_900511755	EuSCAPE_EE007	Homo sapiens	lower respiratory tract secrection	Estonia	2013
GCA_009695525	EN5275	Homo sapiens	blood sample from neonates	India: Kolkata	3-Jan-16
GCA_900493735	4300STDY6470398	Homo sapiens	-	Thailand	2016
GCA_002173875	759	Homo sapiens	-	China:Beijing	2012
GCA_900508905	EuSCAPE_TR229	Homo sapiens	urine	Turkey	2013
GCA_900508915	EuSCAPE_TR231	Homo sapiens	lower respiratory tract secrection	Turkey	2013
GCA_900493725	4300STDY6470400	Homo sapiens	-	Thailand	2016
GCA_900492925	4300STDY6636942	Homo sapiens	-	Thailand	2016
GCA_900494025	4300STDY6470464	Homo sapiens	-	Thailand	2016
GCA_012970645	A16KP0135	Homo sapiens	blood	South Korea: Seoul	2016
GCA_009909305	KUH-KPNHVF1	Homo sapiens	stool	South Korea: Seoul	2017/7/24
GCA_009909325	KUH-KPNHVL1	Homo sapiens	liver abscess	South Korea: Seoul	2017/7/20
GCA_902506975	D4	Homo sapiens	-	China	2011
GCA_901001345	COK193	Homo sapiens	blood	China	2013
GCA_902508885	ZKP149	Homo sapiens	-	China	2017
GCA_901001515	COK301	Homo sapiens	blood	China	2013
GCA_901001965	HAK290	Homo sapiens	blood	China	2013
GCA_901000825	COK43	Homo sapiens	blood	China	2013

GCA_902507145	F1	Homo sapiens	-	China	2012
GCA_900511245	EuSCAPE_AT028	Homo sapiens	wound secretion	Austria	2014
GCA_901001105	COK153	Homo sapiens	blood	China	2013
GCA_902508915	ZKP126	Homo sapiens	-	China	2017
GCA_901001005	COK90	Homo sapiens	blood	China	2013
GCA_902507135	D11	Homo sapiens	-	China	2011
GCA_900494125	4300STDY6470458	Homo sapiens	-	Thailand	2016
GCA_902507025	E6	Homo sapiens	-	China	2012
GCA_001033995	missing	Homo sapiens	-	missing	2013
GCA_900493975	4300STDY6470425	Homo sapiens	-	Thailand	2016
GCA_901001995	HAK278	Homo sapiens	blood	China	2013
GCA_900494055	4300STDY6470459	Homo sapiens	-	Thailand	2016
GCA_900092925	PB94	Homo sapiens	blood	Thailand	2014
GCA_900092945	PB270	Homo sapiens	blood	Thailand	2015
GCA_902506925	B7	Homo sapiens	-	China	2010
GCA_901001565	HAK37	Homo sapiens	blood	China	2013
GCA_901001485	COK309	Homo sapiens	blood	China	2013
GCA_901001715	HAK195	Homo sapiens	blood	China	2013
GCA_901001355	COK191	Homo sapiens	blood	China	2013

GCA_901001705	HAK203	Homo sapiens	blood	China	2013
GCA_002264195	K950	Homo sapiens	blood	China:Jiangxi	9-Jul-14
GCA_901000815	COK71	Homo sapiens	blood	China	2013
GCA_901001405	COK245	Homo sapiens	blood	China	2013
GCA_902508965	ZKP153	Homo sapiens	-	China	2017
GCA_011366155	KP9	Homo sapiens	stool from adult	China:Wenzhou	Mar-17
GCA_901000785	COK8	Homo sapiens	blood	China	2013
GCA_010598135	AS012303	Homo sapiens	lung	USA	2015/7/20
GCA_010602425	AS012302	Homo sapiens	lung	USA	2015/7/20
GCA_900174085	VRCO0171	-	environment	United Kingdom	2015
GCA_900174155	VRCO0173	-	environment	United Kingdom	2015
GCA_900174105	VRCO0178	-	environment	United Kingdom	2015
GCA_900174065	VRCO0170	-	environment	United Kingdom	2015
GCA_900174045	VRCO0167	-	environment	United Kingdom	2015
GCA_900174075	VRCO0172	-	environment	United Kingdom	2015
GCA_900174135	VRCO0176	-	environment	United Kingdom	2015
GCA_900174095	VRCO0177	-	environment	United Kingdom	2015
GCA_900174145	VRCO0175	-	environment	United Kingdom	2015
GCA_900174055	VRCO0169	-	environment	United Kingdom	2015
GCA_900174125	VRCO0174	-	environment	United Kingdom	2015
GCA_902506765	B11	Homo sapiens	-	China	2010
GCA_901001845	HAK207	Homo sapiens	blood	China	2013
GCA_901001905	HAK225	Homo sapiens	blood	China	2013
GCA_901001075	COK129	Homo sapiens	blood	China	2013

GCA_901001085	COK142	Homo sapiens	blood	China	2013
GCA_901001535	COK313	Homo sapiens	blood	China	2013
GCA_901001665	COK292	Homo sapiens	blood	China	2013
GCA_901001895	HAK253	Homo sapiens	blood	China	2013
GCA_901001975	HAK254	Homo sapiens	blood	China	2013
GCA_901001395	COK239	Homo sapiens	blood	China	2013
GCA_901000865	COK40	Homo sapiens	blood	China	2013
GCA_900493905	4300STDY6470422	Homo sapiens	-	Thailand	2016
GCA_902509405	ZKP190	Homo sapiens	-	China	2017
GCA_902508185	ZKP54	Homo sapiens	-	China	2017
GCA_901001595	COK273	Homo sapiens	blood	China	2013
GCA_012970245	C16KP0122	Homo sapiens	blood	South Korea: Gangwon	2016
GCA_900093185	PB502	Homo sapiens	urine	Thailand	2015
GCA_901001325	COK237	Homo sapiens	blood	China	2013
GCA_902507165	F4	Homo sapiens	-	China	2012
GCA_002210445	KpvST23L_OXA-48	Homo sapiens	feces	United Kingdom:London	11-Jan-16
GCA_002813595	SGH10	Homo sapiens	liver abscess	Singapore	unknown
GCA_009887895	VNMU010	Homo sapiens	wound	Ukraine	2014/9/12
GCA_902506775	A10	Homo sapiens	-	China	2009

GCA_902508025	ZKP20	Homo sapiens	-	China	2017
GCA_902508335	ZKP56	Homo sapiens	-	China	2017
GCA_001720635	SCPM-O-B-7850	missing	-	missing	2014
GCA_002114225	ICIS-278_PBV	Homo sapiens	feces	Russia: South Ural	2003
GCA_001033615	missing	Homo sapiens	-	-	2014
GCA_010591245	AS012414	Homo sapiens	lung	USA	2015/1/5
GCA_002761315	P1428	Homo sapiens	sputum	China: Beijing	2007/9/8
GCA_902509365	ZKP187	Homo sapiens	-	China	2017
GCA_901000845	COK78	Homo sapiens	blood	China	2013
GCA_000294365	1084	-	-	-	-
GCA_901000885	COK77	Homo sapiens	blood	China	2013
GCA_902507075	C10	Homo sapiens	-	China	2011
GCA_001707135	UTSW Atlanta 01	Homo sapiens	Femoral bone	USA: Atlanta, Georgia	2015/3/27
GCA_900493965	4300STDY6470424	Homo sapiens	-	Thailand	2016
GCA_001708245	ED2	Homo sapiens	blood	Taiwan	2006
GCA_901001305	COK180	Homo sapiens	blood	China	2013
GCA_902508105	ZKP11	Homo sapiens	-	China	2017
GCA_902508805	ZKP141	Homo sapiens	-	China	2017
GCA_002153255	UCI110	Homo sapiens	-	USA: Massachusetts, Boston	2015

GCA_900515115	EuSCAPE_IT149	Homo sapiens	-	Italy	2014
GCA_902704675	-	-	-	-	-
GCA_001756905	B3789	Homo sapiens	blood	India	2015
GCA_901001795	HAK162	Homo sapiens	blood	China	2013
GCA_901001735	HAK104	Homo sapiens	blood	China	2013
GCA_902508535	ZKP83	Homo sapiens	-	China	2017
GCA_902507235	F5	Homo sapiens	-	China	2012
GCA_901001425	COK248	Homo sapiens	blood	China	2013
GCA_901001435	COK247	Homo sapiens	blood	China	2013
GCA_901000985	COK105	Homo sapiens	blood	China	2013
GCA_901001865	HAK251	Homo sapiens	blood	China	2013
GCA_001034005	missing	Homo sapiens	-	missing	2014
GCA_002369925	1088	Homo sapiens	-	China:Zhejiang	2013/11/30
GCA_901001545	COK294	Homo sapiens	blood	China	2013
GCA_902508715	ZKP107	Homo sapiens	-	China	2017
GCA_901000975	COK85	Homo sapiens	blood	China	2013
GCA_901001875	HAK215	Homo sapiens	blood	China	2013
GCA_901001095	COK135	Homo sapiens	blood	China	2013
GCA_902508755	ZKP113	Homo sapiens	-	China	2017

GCA_901001635	HAK41	Homo sapiens	blood	China	2013
GCA_902506885	A11	Homo sapiens	-	China	2009
GCA_902508225	ZKP49	Homo sapiens	-	China	2017
GCA_901000875	COK81	Homo sapiens	blood	China	2013
GCA_901000925	COK41	Homo sapiens	blood	China	2013
GCA_902508865	ZKP145	Homo sapiens	-	China	2017
GCA_901001455	COK255	Homo sapiens	blood	China	2013
GCA_901001625	COK256	Homo sapiens	blood	China	2013
GCA_002970895	KPHS1249	Homo sapiens	wound	China	2008/6/11
GCA_902508485	ZKP76	Homo sapiens	-	China	2017
GCA_902507945	ZKP16	Homo sapiens	-	China	2017
GCA_901001835	HAK205	Homo sapiens	blood	China	2013
GCA_902508055	ZKP24	Homo sapiens	-	China	2017
GCA_902509325	ZKP188	Homo sapiens	-	China	2017
GCA_902508955	ZKP142	Homo sapiens	-	China	2017
GCA_902509415	ZKP197	Homo sapiens	-	China	2017
GCA_902507125	D10	Homo sapiens	-	China	2011
GCA_901000805	COK68	Homo sapiens	blood	China	2013
GCA_901001295	COK177	Homo sapiens	blood	China	2013

GCA_901001605	COK306	Homo sapiens	blood	China	2013
GCA_901000935	COK80	Homo sapiens	blood	China	2013
GCA_001529935	RJF999	Homo sapiens	blood	China: Shanghai	26-Jan-15
GCA_901001375	COK176	Homo sapiens	blood	China	2013
GCA_901001945	HAK279	Homo sapiens	blood	China	2013
GCA_901000905	COK48	Homo sapiens	blood	China	2013
GCA_901000965	COK110	Homo sapiens	blood	China	2013
GCA_902508165	ZKP45	Homo sapiens	-	China	2017
GCA_901000855	COK70	Homo sapiens	blood	China	2013
GCA_901001065	COK147	Homo sapiens	blood	China	2013
GCA_902508765	ZKP123	Homo sapiens	-	China	2017
GCA_901001055	COK92	Homo sapiens	blood	China	2013
GCA_901000945	COK117	Homo sapiens	blood	China	2013
GCA_902506945	A12	Homo sapiens	-	China	2009
GCA_902508085	ZKP23	Homo sapiens	-	China	2017
GCA_009935715	22	Homo sapiens	cerebrospinal fluid	China: Zhejiang	30-Nov-15
GCA_002206015	329	Homo sapiens	-	China:Beijing	2012
GCA_901001585	HAK26	Homo sapiens	blood	China	2013
GCA_901001495	COK257	Homo sapiens	blood	China	2013

GCA_902508675	ZKP110	Homo sapiens	-	China	2017
GCA_003037185	SCKP020018	Homo sapiens	-	China: Meishan, Sichuan	2016/10/15
GCA_901001855	HAK206	Homo sapiens	blood	China	2013
GCA_002969145	SCPM-O-B-7852	missing	-	missing	2014
GCA_002870905	HS09565	Homo sapiens	-	China:Shanghai	2009/3/11
GCA_002890425	KP1	Homo sapiens	-	China: Shanghai	18-Apr-09
GCA_001939855	GN-3	Homo sapiens	Drainage samples from liver abscess	China:Fifth Affiliated Hospital of Wenzhou Medical University	2015
GCA_002845905	KP9	Homo sapiens	-	China: Shanghai	18-Apr-09
GCA_002870885	LS357	Homo sapiens	Liver abscess puncture fluid	China:Shanghai	14-Mar-12
GCA_003437255	TF06-6	Homo sapiens	feces	China: Shenzhen	2013/7/1
GCA_902506895	B3	Homo sapiens	-	China	2009
GCA_901000795	COK1	Homo sapiens	blood	China	2013
GCA_902509395	ZKP189	Homo sapiens	-	China	2017
GCA_902507035	E5	Homo sapiens	-	China	2012
GCA_901001755	HAK187	Homo sapiens	blood	China	2013
GCA_902507065	C12	Homo sapiens	-	China	2011
GCA_901001025	COK107	Homo sapiens	blood	China	2013

GCA_901001695	HAK120	Homo sapiens	blood	China	2013
GCA_902508625	ZKP108	Homo sapiens	-	China	2017
GCA_902506905	B4	Homo sapiens	-	China	2009
GCA_002851855	WCHKP030209	Homo sapiens	-	China: Sichuan, Chengdu	26-Jun-17
GCA_902508315	ZKP47	Homo sapiens	-	China	2017
GCA_901001015	COK115	Homo sapiens	blood	China	2013
GCA_002853115	WCHKP030925	Homo sapiens	-	China: Sichuan, Chengdu	28-May-16
GCA_901001035	COK101	Homo sapiens	blood	China	2013
GCA_901001235	COK182	Homo sapiens	blood	China	2013
GCA_902507095	E3	Homo sapiens	-	China	2012
GCA_901000835	COK82	Homo sapiens	blood	China	2013
GCA_902507965	ZKP18	Homo sapiens	-	China	2017
GCA_901001275	COK192	Homo sapiens	blood	China	2013
GCA_002831525	KP6	Homo sapiens	-	China: shanghai	18-Apr-09
GCA_002870925	HS102438	Homo sapiens	sputum	China:Shanghai	9-Nov-10
GCA_902509245	ZKP175	Homo sapiens	-	China	2017
GCA_902508695	ZKP105	Homo sapiens	-	China	2017
GCA_901001415	COK241	Homo sapiens	blood	China	2013
GCA_901001825	HAK147	Homo sapiens	blood	China	2013

GCA_901001935	HAK295	Homo sapiens	blood	China	2013
GCA_902508745	ZKP115	Homo sapiens	-	China	2017
GCA_902507085	D8	Homo sapiens	-	China	2011
GCA_901001465	COK178	Homo sapiens	blood	China	2013
GCA_002264435	K5065	Homo sapiens	blood	China:Zhejiang	21-Oct-15
GCA_902509235	ZKP185	Homo sapiens	-	China	2017
GCA_901001365	COK199	Homo sapiens	blood	China	2013
GCA_902507015	D3	Homo sapiens	-	China	2011
GCA_902508275	ZKP55	Homo sapiens	-	China	2017
PRJNA685215	C11	Homo sapiens	-	China	2014
PRJNA685215	C15	Homo sapiens	-	China	2014
PRJNA685215	C7	Homo sapiens	-	China	2014
PRJNA685215	C9	Homo sapiens	-	China	2014
PRJNA685215	Kpn121	Homo sapiens	-	China	2009
PRJNA685215	Kpn335	Homo sapiens	-	China	2010
PRJNA685215	KPN5320	Homo sapiens	-	China	2017
PRJNA685215	Z108	Homo sapiens	-	China	2018
PRJNA685215	Z109	Homo sapiens	-	China	2018
PRJNA685215	Z201	Homo sapiens	-	China	2018

SRR5893961	Kpn8	Homo sapiens	-	China	2009
SRR5893944	Kpn25	Homo sapiens	-	China	2013
SRR5893947	Kpn28	Homo sapiens	-	China	2013
SRR5893954	Kpn30	Homo sapiens	-	China	2013
SRR5893980	Kpn37	Homo sapiens	-	China	2013
SRR5893955	Kpn41	Homo sapiens	-	China	2013
SRR5893942	Kpn43	Homo sapiens	-	China	2013
PRJNA575579	Kpn355	Homo sapiens	-	China	2019
PRJNA575579	Kpn360	Homo sapiens	-	China	2019
PRJNA575579	KPN-T46	Homo sapiens	-	China	2019
PRJNA575579	BDMS190620071	Homo sapiens	-	China	2019
PRJNA575579	BDMS190620072	Homo sapiens	-	China	2019
PRJNA575579	BDMS190620084	Homo sapiens	-	China	2019
PRJNA575579	BDMS190620085	Homo sapiens	-	China	2019
PRJNA575579	BDMS190620086	Homo sapiens	-	China	2019
PRJNA575579	BDMS190620087	Homo sapiens	-	China	2019
PRJNA575579	BDMS190620088	Homo sapiens	-	China	2019
PRJNA575579	BDMS190620089	Homo sapiens	-	China	2019

-: the information is not available.

Table S3. Presence of key virulence genes in hvKp

Virulence gene	Non MDR hvKp (n=205)	MDR hvKp (n=34)	P value
iucA	205 (100.0%)	34 (100.0%)	-
iroB	194 (94.6%)	26 (76.5%)	0.001024
peg-344	181 (88.3%)	31 (91.2%)	0.8419
peg-589	205 (100.0%)	34 (100.0%)	-
rmpA	173 (84.4%)	26 (76.5%)	0.3694
rmpA2	60 (29.3%)	11 (32.4%)	0.8714

Table S4. Correlation of plasmid Inc types with the number of resistance classes in *K. pneumoniae* ST23 genomes

Inc type	No. of strains of different resistance classes (n [%])		P
	<3 (total=212)	>=3 (total =34)	
IncHI1B	203 (95.3)	27 (79.4)	0.0036
IncFII	25 (11.7)	25 (73.5)	<0.001
IncFIB	23 (10.8)	10 (29.4)	0.0063
IncR	7 (3.3)	6 (17.6)	0.0037
ColRNAI	6 (2.8)	5 (14.7)	0.0093
IncFIA	4 (1.9)	4 (11.8)	0.014
IncA/C2	0 (0.0)	3 (8.8)	0.0024
IncN	0 (0.0)	3 (8.8)	0.0024
IncL/M	3 (1.4)	3 (8.8)	0.036
Col156	5 (2.3)	3 (8.8)	0.08
IncX3	3 (1.4)	2 (5.9)	0.14
ColKP3	6 (2.8)	2 (5.9)	0.30
IncI1	0 (0.0)	1 (2.9)	0.14
IncX1	0 (0.0)	1 (2.9)	0.14
IncQ1	1 (0.5)	1 (2.9)	0.26
ColpVC	4 (1.9)	1 (2.9)	0.53
Col	7 (3.3)	1 (2.9)	1.00
Col3M	2 (0.9)	0 (0.0)	1.00

Table S5. Genomic islands encoding resistance genes identified in the 246 genomes.

Strain	Contig	Contig length	Start	End	Length	ARGs	ISs
GCA_001720635	GCA_001720635_90	9,289	1,322	8,390	7,069	*aadB, sul1*	IS1326, IS1353
GCA_001902335	GCA_001902335_5	81,641	35,176	56,748	21,573	*qnrS1, catA2, sul2, tet(A)*	IS26, IS5075, ISVsa3
GCA_001902335	GCA_001902335_6	63,588	34,428	40,093	5,666	*blaOXA-48*	IS10A, IS1R
GCA_001902335	GCA_001902335_7	57,266	1,832	3,673	1,842	*blaCTX-M-79*	IS26
GCA_001902335	GCA_001902335_7	57,266	38,693	56,295	17,603	*aac(3)-IIa, aac(6')Ib-cr, blaOXA-1*	IS1A, IS1R, IS26, ISKpn11
GCA_001970155	GCA_001970155_62	8,412	2,406	6,508	4,103	*sul2*	IS5075, ISVsa3
GCA_001970155	GCA_001970155_74	2,854	128	2,684	2,557	*aac(3)-IIa*	ISKpn11
GCA_002163895	GCA_002163895_2	230,606	57,916	78,314	20,399	*qnrB4, blaDHA-1, blaSHV-12, sul1*	IS26, ISCR1
GCA_002210445	GCA_002210445_20	69,086	1,046	6,711	5,666	*blaOXA-48*	IS10A, IS1R

GCA_002210445	GCA_002210445_20	69,086	64,545	67,473	2,929	*blaOXA-48*	IS10A, IS1R
GCA_002264195	GCA_002264195_19	48,964	6,750	12,328	5,579	*blaNDM-1*	IS3000, IS5
GCA_002264435	GCA_002264435_26	10,754	607	2,848	2,242	*blaKPC-2*	ISKpn27
GCA_002369925	GCA_002369925_2	52,921	18,078	38,272	20,195	*blaCTX-M-55, blaTEM-1B, blaTEM-209, rmtB*	IS1294, IS26
GCA_002369925	GCA_002369925_4	105,071	21,331	33,280	11,950	*blaKPC-2*	IS26, IS5075, ISKPn14, ISKpn27
GCA_002969145	GCA_002969145_16	7,060	1,099	3,678	2,580	*blaCTX-M-15*	ISEcp1
GCA_003037185	GCA_003037185_39	49,628	6,750	12,992	6,243	*blaNDM-1*	IS3000, IS5
GCA_003037185	GCA_003037185_73	4,195	1,433	3,606	2,174	*blaCTX-M-15*	IS1X2
GCA_009661575	GCA_009661575_24	45,473	42,625	45,255	2,631	*catA2*	IS26
GCA_009661655	GCA_009661655_31	8,411	1,905	6,007	4,103	*sul2*	IS5075, ISVsa3
GCA_009661655	GCA_009661655_45	2,852	170	2,726	2,557	*aac(3)-IIa*	ISKpn11

GCA_009695525	GCA_009695525_87	11,908	1,639	11,310	9,672	*armA, mph(E), msr(E), sul1*	ISCR1, ISEc28, ISEc29
GCA_009695525	GCA_009695525_89	10,857	423	8,216	7,794	*blaCTX-M-15, blaTEM-1B, strA, strB, sul2*	ISEcp1
GCA_009935715	GCA_009935715_1	83,145	20,403	24,024	3,622	*blaKPC-2*	ISKPn14, ISKpn27
GCA_012970245	GCA_012970245_3	51,930	5,069	12,337	7,269	*blaCTX-M-14*	IS6100, ISEcp1
GCA_012970245	GCA_012970245_3	51,930	30,051	32,554	2,504	*qnrS1*	IS26
GCA_012970645	GCA_012970645_3	85,040	7,158	14,069	6,912	*qnrS1, blaCTX-M-15, blaTEM-1B*	ISEcp1
GCA_900092925	GCA_900092925_42	12,615	4,078	12,615	8,538	*qnrS1, blaCTX-M-55*	IS26
GCA_900092945	GCA_900092945_25	12,272	646	7,989	7,344	*qnrS1, blaCTX-M-55*	IS1SD
GCA_900093185	GCA_900093185_18	7,674	168	4,210	4,043	*mph(A)*	IS6100
GCA_900093185	GCA_900093185_45	25,354	12,125	17,899	5,775	*aac(3)-IId, blaCTX-M-15, blaTEM-1C*	ISEcp1
GCA_900093185	GCA_900093185_57	11,817	1,549	11,639	10,091	*ARR-3, aac(6')-Iaf, aac(6')Ib-cr, aadA1, aadA16, dfrA27, tet(A)*	IS26

GCA_900093185	GCA_900093185_61	4,423	481	4,131	3,651	*qnrB6*	ISCR1
GCA_900174045	GCA_900174045_9	76,554	14,402	16,317	1,916	*blaTEM-1B*	IS26
GCA_900174055	GCA_900174055_13	18,121	15,696	17,611	1,916	*blaTEM-1B*	IS26
GCA_900174065	GCA_900174065_17	78,482	14,473	16,388	1,916	*blaTEM-1B*	IS26
GCA_900174075	GCA_900174075_15	52,817	50,902	52,817	1,916	*blaTEM-1B*	IS26
GCA_900174085	GCA_900174085_10	7,642	438	7,493	7,056	*mph(A), sul1*	IS6100
GCA_900174095	GCA_900174095_40	120,901	58,785	60,700	1,916	*blaTEM-1B*	IS26
GCA_900174105	GCA_900174105_30	62,774	60,349	62,264	1,916	*blaTEM-1B*	IS26
GCA_900174125	GCA_900174125_4	74,783	12,667	14,582	1,916	*blaTEM-1B*	IS26
GCA_900174135	GCA_900174135_14	185,935	60,271	62,186	1,916	*blaTEM-1B*	IS26
GCA_900174145	GCA_900174145_12	30,278	15,697	17,612	1,916	*blaTEM-1B*	IS26

GCA_900174155	GCA_900174155_58	8,381	149	8,381	8,233	*mph(A), sul1*	IS26, IS6100
GCA_900493905	GCA_900493905_41	8,663	361	4,601	4,241	*blaTEM-1B*	IS26
GCA_900493905	GCA_900493905_63	2,940	242	2,209	1,968	*aac(3)-IId*	ISVsa5
GCA_900493905	GCA_900493905_9	10,216	195	8,585	8,391	*aadA5, mph(A), sul1*	IS6100
GCA_901001455	GCA_901001455_89	51,208	45,858	48,431	2,574	*blaCTX-M-14*	ISEcp1
GCA_901001495	GCA_901001495_267	84,777	19,750	22,329	2,580	*blaCTX-M-15*	ISEcp1
GCA_901001625	GCA_901001625_24	48,663	45,946	48,519	2,574	*blaCTX-M-14*	ISEcp1
GCA_901001755	GCA_901001755_1995	51,212	48,494	51,067	2,574	*blaCTX-M-14*	ISEcp1
GCA_901001935	GCA_901001935_168	2,653	265	2,522	2,258	*aph(3')-IIa*	IS50R
GCA_901001935	GCA_901001935_323	5,203	91	4,133	4,043	*mph(A)*	IS6100
GCA_901001935	GCA_901001935_532	4,702	403	4,389	3,987	*qnrB52*	ISCR1

GCA_901001935	GCA_901001935_721	6,908	1,820	6,887	5,068	*mph(E), msr(E)*	ISEc28, ISEc29
GCA_901001995	GCA_901001995_5433	19,854	1,402	13,954	12,553	*qnrB4, aadA2, blaDHA-1, dfrA12, sul1*	ISCR1
GCA_902507135	GCA_902507135_11	13,339	692	13,252	12,561	*ant(3'')-Ih-aac(6')-IId, blaCTX-M-14, cmlA1, mph(A)*	IS6100, ISEcp1
GCA_902508185	GCA_902508185_60	45,828	265	4,207	3,943	*aph(3')-IIa*	IS1294, IS50R
GCA_902508225	GCA_902508225_35	6,600	903	6,044	5,142	*strA, strB*	IS1133, ISEc8
Kpn25	Kpn25_20	109,021	76,727	98,196	21,470	*qnrB4, blaCTX-M-14, blaDHA-1, sul1*	IS26, IS6100, IS903B, ISCR1
Kpn43	Kpn43_24	98,274	76,622	98,091	21,470	*qnrB4, blaCTX-M-14, blaDHA-1, sul1*	IS26, IS6100, IS903B, ISCR1

Table S6. Characteristics of ST23 MDR-HvKp sequenced by both next-generation and third-generation sequencing.

**Characteristics**	**Kpn5320**	**KpnT46**
Patient	Female, 66 y	Female, 42 y
Hospital, time	A, 2017	B-TS, 2018
Sample	Abscess	Sputum
Infection type	Community-acquired infection	Hospital-acquired infection
Department	Hepatobiliary Surgery	ICU
Underlying disease	Cancer	Digestive diseases, Cerebrovascular disease, Surgery within 90 days
Antibiotics pre-exposure within 90 days	+	+
Metastatic infection	-	-
Central intravenous catheter	-	+
Urinary tube	-	+
Endotracheal tube	-	+
Urinary catheter	-	+
Gastrostomy tube	-	+
Drainage tube	-	+
SOFA > 6	-	-
CCI > 4	-	-
Septic shock	-	+

Monotherapy	+	+
Switching antibiotics	+	-
Outcomes within 30 days	Survived	Survived
Non sensitive to antibiotics	Tigecycline, Ceftriaxone, Cefazolin, Minocycline, Ampicillin/Sulbactam	Cefazolin, Ampicillin/Sulbactam, Ceftazidime, Aztreonam
Hypermucoviscosity	+	+
Hypervirulence	+	+
Genotype	KL1-ST23-O1v2	KL1-ST23-O1v2
Plasmid(n)	2	2
Resistance replication	IncL/M	IncFII
Resistance marker	bla_{TEM-1B} , $bla_{CTX-M-3}$	*qnrS1, tet(A),* bla_{TEM-1B}
Virulence replication	IncHI1B	IncHI1B
Capsule regulator	*rmpA+rmpA2*	*rmpA+rmpA2*
Yersiniabactin-ST	YbST46-ICEKp10	YbST46-ICEKp10
Colibactin-ST	CbST29:	CbST29
Aerobactin-ST	AbST1: *iucA1B1C1D1iutA1*	AbST1: *iucA1B1C1D1iutA1*
Salmochelin-ST	SmST2-1LV: iroB1C4*D1N1	SmST2: iroB1C4D1N1

Table S7. Plasmid fragments encoding resistance genes identified in the 246 genomes.

Contig	Contig length	Plasmid genes	Plasmid Inc	ISs	Resistant_genes
GCA_900174085_12	102,689	traA, traC, traD, traI, traJ, traM, traQ, traV	Col156|IncFIB|IncFII	IS100kyp, IS21	*blaTEM-1B*
GCA_900174125_4	74,783	traA, traC, traD, traI, traJ, traM, traQ, traV	Col156|IncFII	IS26	*blaTEM-1B*
GCA_900174145_12	30,278	traD, traI	Col156|IncFII	IS26	*blaTEM-1B*
GCA_900174135_14	185,935	repB, traA, traC, traD, traI, traJ, traM, traQ, traV	IncFIB|IncFII	IS21, IS26	*blaTEM-1B*
GCA_900174095_40	120,901	repB, traA, traC, traD, traI, traJ, traM, traQ, traV	IncFIB|IncFII	IS100kyp, IS26, ISEhe3	*blaTEM-1B*
GCA_900174065_17	78,482	traA, traC, traD, traI, traJ, traM, traQ, traV	IncFIB|IncFII	IS21, IS26	*blaTEM-1B*
GCA_900174045_9	76,554	traA, traC, traD, traI, traJ, traM, traQ, traV	IncFIB|IncFII	IS21, IS26	*blaTEM-1B*
GCA_900174105_30	62,774	traA, traC, traI, traJ, traM, traQ, traV	IncFII	IS26	*blaTEM-1B*
GCA_900174075_15	52,817	traA, traC, traD, traI, traJ, traM, traQ, traV	IncFII	IS26	*blaTEM-1B*

GCA_900174155_45	52,092	traA, traC, traD, traI, traJ, traM, traQ, traV	IncFII	-	*blaTEM-1B*
KPN-T46_3	121,145	repA, traA, traC, traD, traI, traJ, traM, traQ, traS, traV	IncFII|IncQ1	IS1X2, IS26, IS26, IS26, ISKpn24, ISSoEn2, ISSwi1	*qnrS1, blaCTX-M-3, blaTEM-1B, floR, strA, strB, sul2, tet(A)*
BDMS190620084-1a_16	137,449	repE, traA, traC, traD, traI, traJ, traM, traQ, traV	IncFII|IncR	IS1X2, IS26, IS6100, IS903B, ISCR1, ISKpn24	*qnrB4, blaCTX-M-14, blaDHA-1, sul1*
BDMS190620088-1a_17	133,571	repE, traA, traC, traD, traI, traJ, traM, traQ, traV	IncFII|IncR	IS1X2, IS26, IS6100, IS903B, ISCR1, ISKpn24	*qnrB4, blaCTX-M-14, blaDHA-1, sul1*
Kpn25_20	109,021	repE, traA, traC, traD, traI, traJ, traM, traQ, traV	IncFII|IncR	IS1X2, IS26, IS6100, IS903B, ISCR1, ISKpn24	*qnrB4, blaCTX-M-14, blaDHA-1, sul1*
Kpn43_24	98,274	traA, traC, traD, traI, traJ, traM, traQ, traV	IncFII	IS1X2, IS26, IS6100, IS903B, ISCR1, ISKpn24	*qnrB4, blaCTX-M-14, blaDHA-1, sul1*
GCA_901001455_89	51,208	traA, traC, traD, traI, traJ, traM, traQ, traV	IncFII	ISEcp1	*blaCTX-M-14*

GCA_901001625_24	48,663	traA, traC, traD, traI, traJ, traM, traQ, traV	IncFII	ISEcp1	*blaCTX-M-14*
GCA_012970645_3	85,040	repA, traA, traC, traD, traI, traJ, traM, traQ, traS, traV, virB	IncFIB|IncFII	ISEcp1	*qnrS1, blaCTX-M-15, blaTEM-1B*
GCA_902508185_60	45,828	traA, traC, traD, traI, traJ, traM, traQ, traS, traV	IncFII	IS1294, IS50R	*aph(3')-IIa*
GCA_001902335_7	57,266	traA, traC, traJ, traM, traV	IncFII	IS1A, IS1R, IS26, IS26, IS26, IS26, ISEc23, ISKpn11	*aac(3)-IIa, aac(6')Ib-cr, blaCTX-M-79, blaOXA-1*
GCA_901001995_3883	54,560	traC, traD, traI, traQ, traS, traV	IncFII	-	*tet(A)*
GCA_002369925_2	52,921	traI	IncFII	IS1294, IS26, IS26, IS26, IS26	*blaCTX-M-55, blaTEM-1B, blaTEM-209, rmtB*
Kpn37_32	45,987	repA	IncFII	-	*strA, strB*
GCA_901001495_267	84,777	repA, traC, traI	IncI1	ISEcp1	*blaCTX-M-15*
KPN5320_3	74,311	repA, traC, traI	IncL/M	IS26, IS26, ISEcp1	*aac(3)-IId, blaCTX-M-3, blaTEM-1B*

GCA_002210445_20	69,086	repA, traC, traI	IncL/M	IS10A, IS10A, IS10A, IS1R, IS1R, IS1R	*blaOXA-48, blaOXA-48*
GCA_001902335_6	63,588	repA, traC, traI	IncL/M	IS10A, IS10A, IS1R, IS1R	*blaOXA-48*
GCA_012970245_3	51,930	repE, traD, traI, virB1, virB4, virB5, virB6, virB8, virB9	IncN	IS26, IS6100, ISEcp1	*qnrS1, blaCTX-M-14*
GCA_003037185_39	49,628	virB1, virB4, virB8, virB9, virD4	IncX3	IS3000, IS5	*blaNDM-1*
GCA_002264195_19	48,964	virB1, virB4, virB8, virB9, virD4	IncX3	IS3000, IS5	*blaNDM-1*
